# Glomerulonephritis and granulomatous vasculitis in kidney as a complication of the use of BRAF and MEK inhibitors in the treatment of metastatic melanoma: A case report

**DOI:** 10.1097/MD.0000000000007196

**Published:** 2017-06-23

**Authors:** Mehdi Maanaoui, Camille Saint-Jacques, Viviane Gnemmi, Marie Frimat, Arnaud Lionet, Marc Hazzan, Christian Noël, François Provot

**Affiliations:** a Department of Nephrology, CHU Lille, University of Lille, Lille, France; b Department of Pathology, University of Lille, Inserm, CHU Lille, UMR-S 1172 – JPARC – Jean-Pierre Aubert Research Center, Lille, France.

**Keywords:** BRAF, glomerulonephritis, kidney, melanoma, vasculitis

## Abstract

**Rationale:**

BRAF and MEK inhibitors have significantly improved the prognosis of metastatic melanoma, by inhibiting both the mitogen-activated protein kinase (MAP-kinase) pathway. They are associated with infrequent adverse kidney events. Most of these are related to the use of BRAF inhibitors and involve interstitial nephritis with acute tubular necrosis.

**Patient concerns:**

We report a unique case of glomerulonephritis with renal granulomatous vasculitis in a patient diagnosed with metastatic melanoma treated with BRAF and MEK inhibitors. The patient was a 55-year old woman, who presented a melanoma of the right thigh with pulmonary metastasis. Treatment started in November 2015, with Encorafenib and Binimetinib, new BRAF and MEK inhibitors, respectively. Two months after the beginning of the treatment, there was a worsening of her renal function with significant proteinuria.

**Diagnoses:**

Kidney biopsy showed extracapillary proliferation in the glomeruli with a granulomatous reaction.

**Interventions and outcomes:**

Renal function recovered completely after withdrawal of the chemotherapy.

**Lessons:**

All the reported kidney adverse events secondary to BRAF and MEK inhibitors in the literature are related to the use of BRAF inhibitors. Some previous reported mechanistic investigations also provide insight between BRAF inhibitors and podocytes injuries. Therefore, encorafenib most likely is the main responsible of the disease. However, evidence has emerged that inhibition of the MAP kinase pathway could also enhance autoimmunity. Thus, binimetinib may also have played a role and the combination of BRAF and MEK inhibitors may have facilitated this autoimmune kidney disease.

## 1 Introduction

BRAF and MEK inhibitors have significantly changed the prognosis of metastatic melanoma, increasing the period of survival by months. In carcinoma cells, they act upon the mitogen-activated protein kinase (MAP-kinase) pathway, which is essential for cell proliferation and survival. BRAF inhibitors induce a complete blockade of the MAP-kinase pathway, necessary for cell death. However, emergence of BRAF inhibitors resistance can happen quickly after the beginning of the treatment. Thus, MEK inhibitors, by targeting synergistically the MAP-kinase pathway, help maintaining a full MAP-kinase inhibition and a longer treatment efficiency.^[1]^ In January 2016, the Cancer and Kidney International Network reviewed all reports on kidney injury resulting from the use of BRAF inhibitors,^[2]^ especially vemurafenib and dabrafenib. Most of the cases described reported interstitial nephritis with acute tubular necrosis; hence, it was recommended to monitor serum creatinine while using these agents. In February 2017, Perico et al^[3]^ reported the first case of nephrotic syndrome in a patient treated with dabrafenib for a metastatic melanoma. We describe a unique case of glomerulonephritis with renal granulomatous vasculitis secondary to the use of BRAF and MEK inhibitors.

## 2 Case presentation

A 55-year-old woman was hospitalized in the nephrology unit of Huriez Hospital, Lille, in January 2016. She had no previous history of any major disease. She had been diagnosed a superficial spreading type melanoma of the right thigh in March 2015, with BRAF V600E mutation. In September 2015, a CT-scan detected a pulmonary metastasis. She was then treated with encorafenib (450 mg once a day per os), a new BRAF inhibitor, and binimetinib (45 mg twice a day per os), a MEK inhibitor.

The treatment started in November 2015, when serum creatinine concentration was 0.77 mg/dL. In January, the laboratory testing measured a serum creatinine concentration of 2.8 mg/dL, prompting transfer to our nephrology department. On arrival the patient's BP was 130/70 mm Hg, and her heart rate and temperature were 88 bpm and 37.6 °C, respectively. She weighed 74 kg. She only complained of having experienced joint pain in the previous few weeks, but examination revealed no arthritis. Otherwise, examination results were completely normal. She did not present any rash or skin lesions on the previous days. Her recent medical history did not record new events. Three days before she arrived, she took ibuprofen 200 mg twice a day. She did not take any other medication.

The patient's serum creatinine concentration was 2.8 mg/dL, with blood urea 114 mg/dL, sodium level 133 mmol/L, and potassium level 5 mmol/L. Albumin level was 33 g/L and calcium level 8.4 mg/dL. C-reactive protein level was 1.23 mg/dL. She had a leucocyte count of 11,000/mm^3^ including 8700 polynuclear neutrophils and 1500 lymphocytes without polynuclear eosinophils.

Urine analysis showed a 1 g/day proteinuria, without leucocyturia or hematuria.

Serum protein electrophoresis was normal. Plasma tests for antineutrophil cytoplasm antibody and antiglomerular basement membrane antibody were negative. The test for antinuclear antibodies was negative.

A kidney biopsy was performed. Light microscopy revealed 6 glomeruli, including one that was globally sclerotic, with endocapillary proliferation in half of them. Four showed extracapillary proliferation with a granulomatous reaction. Several arterioles exhibited acute necrotizing arteritis with fibrinoid necrosis and a perivascular infiltrate that had a granulomatous appearance with palisading epithelioid macrophages. Major tubular necrosis was also present. Immunofluorescence was weakly positive for C1q and C3 staining, with focal and segmental endomembranous deposits. It was strongly positive for fibrinogen in the crescents. Immunostaining for kappa, lambda, IgG, IgA, and IgM was negative.

Electron microscopy, in one glomerulus without crescent, showed podocytes with cytoplasmic swelling and vacuolization. There was also focal interdigitating foot-process effacement. We did not find any debris or deposit in the subendothelial space.

Encorafenib and binimetinib were then stopped the 5th of January. The patient's serum creatinine decreased subsequently to 1.5 mg/dL at the beginning of February with a proteinuria stable at 1.2 g/24 hour. The patient did not receive steroids.

Starting March 2016, she was subsequently treated with pembrolizumab, an immunotherapy targeting program cell death 1. From March to July 2016, serum creatinine level was around 1.1 mg/dL, and there was a resolution of proteinuria. However, there was neither improvement nor worsening of the cancer lesions with pembrolizumab. Therefore, in July 2016 dabrafenib and trametinib, alternative BRAF and MEK inhibitors were introduced, and pembrolizumab was stopped. In October 2016, serum creatinine was 1.0 mg/dL and urine analysis showed no proteinuria.

## 3 Discussion and conclusion

To our knowledge, this is the first case of glomerulonephritis with granulomatous vasculitis related to the use of new BRAF and MEK inhibitors, encorafenib and binimetinib. The patient completely recovered after the withdrawal of the treatment, without recourse to corticosteroids or other immunosuppressive treatment: the proteinuria and creatinine decreased quickly after stopping the chemotherapy. We could exclude a paraneoplastic glomerular disease as a cause since the cancer did not develop at that time. We also excluded the ibuprofen as a cause of the glomerulonephritis. First, she experienced joints pain before taking the ibuprofen, and we believe that these pains are part of the vasculitis disease. Second, the short time between she first took the medication and the discovery of her renal disease eliminates the ibruprofen as responsible of the crescentic glomerulonephritis with vasculitis. Therefore, we think that either BRAF or MEK inhibitors are responsible of the disease. BRAF and MEK inhibitors both inhibit the MAP kinase pathway.^[4]^ In the literature, most reported kidney adverse events associated with BRAF or MEK inhibitors are related to the use of BRAF inhibitors, especially vemurafenib and dabrafenib. The Food and Drug Administration approved them for clinical use in 2011 and 2013, respectively, which makes them currently well-established therapies. Between 2011 and 2015, around 150 cases of acute kidney injury have been reported for these 2 medicines.^[5]^ Only a few kidney biopsies were reported, which showed acute tubular necrosis and interstitial fibrosis.^[6–7]^ No glomerular injuries were reported. However, according to the Cancer and Kidney International Network, one case of granulomatous nephritis was mentioned in the summary product report of dabrafenib.^[2]^

No adverse renal events have been reported with MEK inhibitors.

We found only 1 case similar to ours. Mirouse et al^[8]^ described the case of a 75-year-old man with a history of Erdheim–Chester disease, treated with vemurafenib. Two years after treatment he was hospitalized for diffusive edema with acute kidney injury. He had no significant proteinuria. Kidney biopsy showed pauci-immune crescentic glomerulonephritis. Renal function recovered completely after cyclophosphamide and high-dose steroid treatment. Mirouse et al^[8]^ also reviewed all the cases reporting vasculitis while using vemurafenib and found 5 other cases. All these cases presented cutaneous effects of vemurafenib, with skin vasculitis. No kidney injuries were reported in these cases.

What supports our hypothesis of BRAF inhibitors as the main responsible of the adverse effect is that Perico et al^[3]^ presented in February 2017 the first report of a nephrotic syndrome owing to dabrafenib treatment. It was the case of a 65-year-old woman, treated with dabrafenib and trametinib for a metastatic melanoma of the right thigh. She presented diffusive edema 8 months after the beginning of the treatment. Proteinuria was 4 g/24 hour. Kidney biopsies and scanning electron microscopy showed severe injuries on the podocytes: podocyte swelling and extensive interdigitating foot process effacement, and also a diffuse thickness of the glomerular basement membrane. Like our case, the glomerular injuries recovered with the interruption of the chemotherapy, without any immunosuppressive treatment. Then, Perico et al^[3]^ provided strong evidence for the involvement of dabrafenib in the podocyte injuries. Phospholipase C epsilon 1 (PLCε1) was already known to interact directly with BRAF in the podocytes.^[9]^ They stained kidney biopsies before and after stopping the chemotherapy for PLCε1 and found that PLCε1 was less present while using dabrafenib. They also tested in vitro the effect of dabrafenib on cultured human podocytes and showed that it downregulated nephrin expression and increased the permeability for albumin of podocyte monolayers. Given the role of PLCε1 mutations involvement in nephrotic syndrome,^[10]^ this report provides a direct connection between the BRAF inhibitors to the glomerular disease. However, vasculitis and crescentic glomerulonephritis probably involve more mechanisms than only the direct toxicity of the BRAF inhibitor on podocytes, for example, a strong immune reaction with an inappropriate inflammatory response may be involved as well.^[11]^ Most reports on drug-induced vasculitis show the presence of antineutrophil cytoplasm antibody or antiglomerular basement membrane antibody secondary to the use of the drug.^[12]^ The hypothesis is that these drugs uncover masked antigens, which then induce an autoimmune reaction. BRAF and MEK inhibitors both inhibit the MAPK pathway and the ERK production. The ERK pathway is known as a key in the pathogenesis of lupus.^[13]^ Lupus TCD4 cells present a defect of the ERK pathway, which causes abnormal epigenetic modifications that could be responsible for autoimmunity.^[13]^ What is more, inhibition of the ERK pathway stimulates autoreactivity in vitro and autoimmunity in vivo.^[14]^ These findings indicate that inhibition of the ERK pathway could enhance an autoimmune reaction. In our case no autoantibodies were detected. We believe that BRAF inhibitors, through the BRAF- PLCε1 pathway, induced in our patient podocyte injuries and uncovered masked antigens to the immune system. The combined inhibition of the MAPK pathway by BRAF and MEK inhibitors may have facilitated an autoimmune response against these antigens, which would explain why our patient did not only have podocyte injuries but also a massive inflammation in the glomeruli. There may have been an autoimmune antibody that we have not detected.

In this case report, we present evidence that the combination of BRAF and MEK inhibitors, and especially in our case encorafenib and binimetinib, may cause glomerular injuries and granulomatous vasculitis with glomerulonephritis.

**Figure 1:**
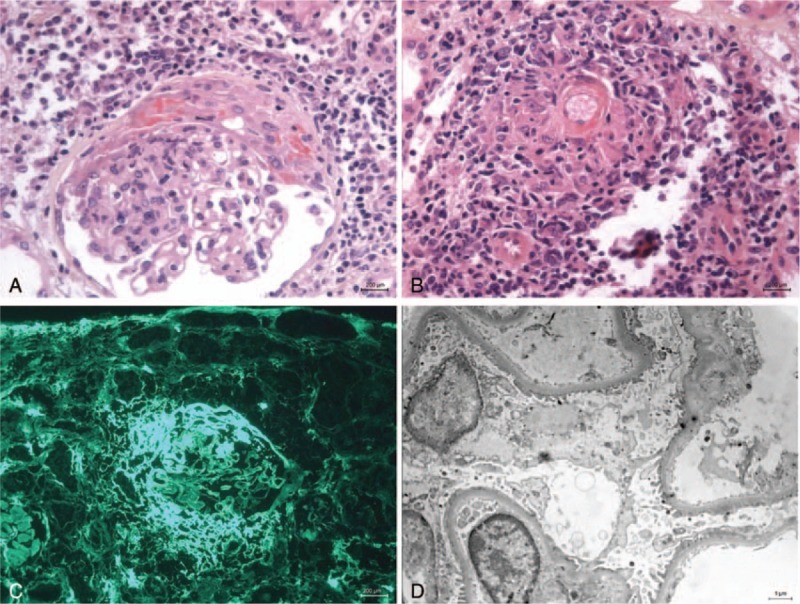
Higher-magnification photomicrographs of the renal biopsy specimen from the patient with metastatic melanoma treated with BRAF inhibitors showing features of crescentic glomerulonephritis associated with podocytes injury. (A) Well-formed cellular crescent with inflammatory cells in some capillaries and segmental fibrinoid necrosis that stains red with hematoxylin-eosin-saffron (HES) stain. There is a diffuse tubulo-interstitial inflammatory infiltrate adjacent to the necrotic portion of the glomerular tuft stained with original magnification, ×400; (B) necrotizing arteritis affecting an arteriole: well-defined surround granulomatous inflammation with palisading epithelioid macrophage is seen. HES stain; original magnification, ×200; (C) immunofluorescence microscopy showing staining for fibrinogen in a large circumferential crescent. Original magnification, ×400; (D) transmission electron micrograph showing interdigitating foot-process effacement with cytoplasmic swelling and vacuolization of podocytes. Original magnification, ×2156.

**Figure 2:**
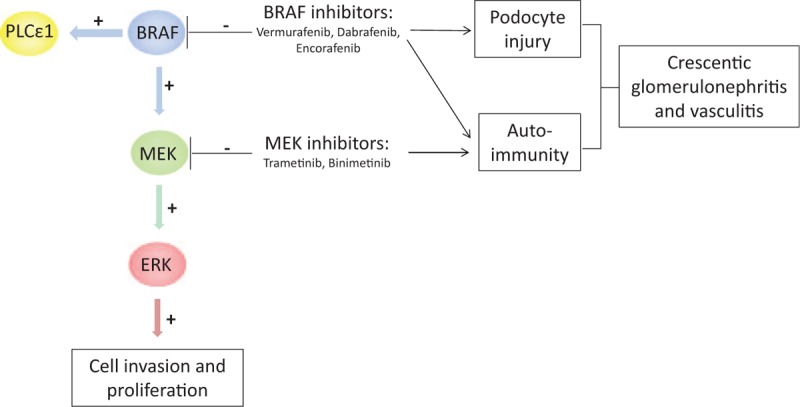
BRAF and MEK inhibitors both inhibit the MEK-ERK pathway. This combination can therefore stimulate autoimmunity. BRAF inhibitors also target specifically the podocytes and inhibit phospholipase C epsilon 1 (PLCε1).

**Figure 3:**
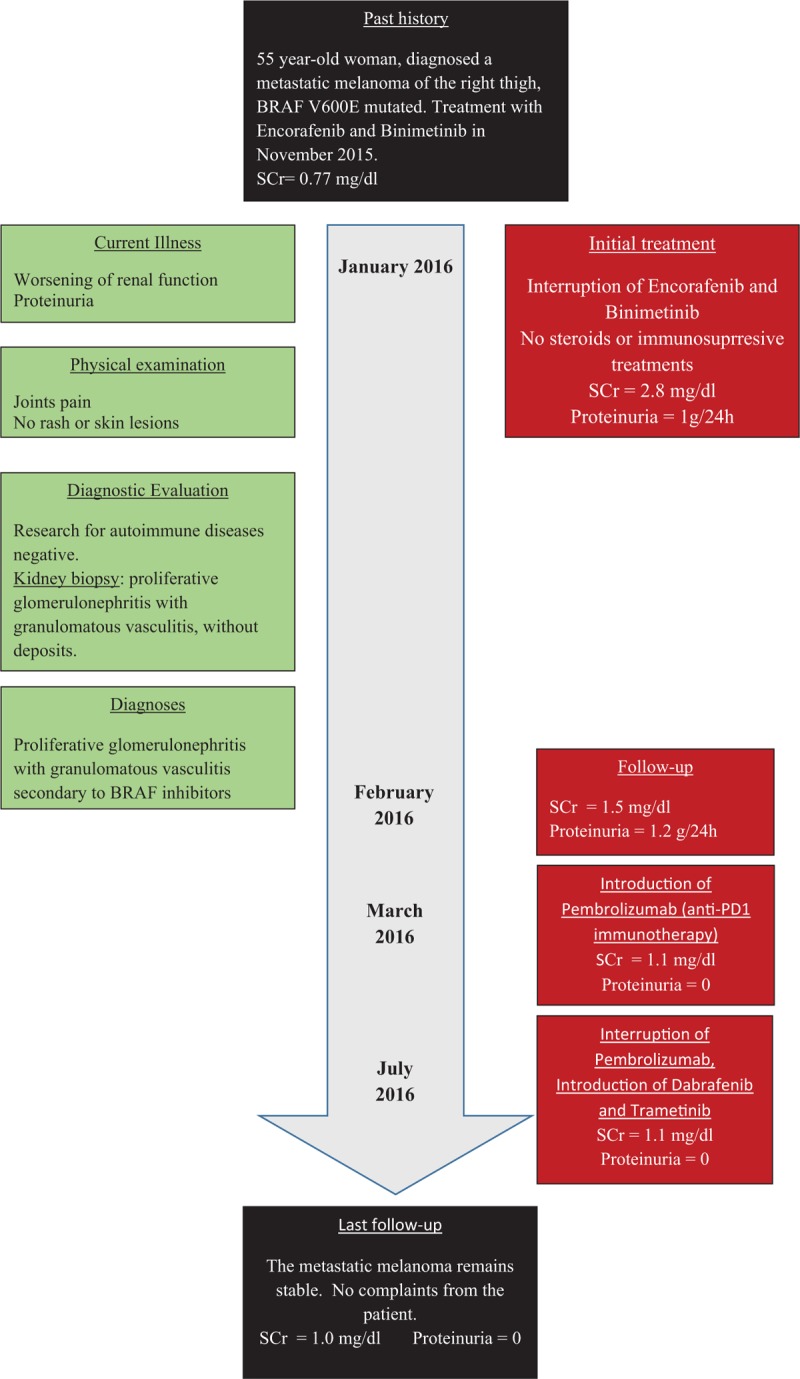
Timeline.

An important point to note is that, after the reintroduction of other BRAF and MEK inhibitors, dabrafenib and trametinib, there was no further deterioration. Therefore, our findings indicate that if glomerular injuries occur while using 1 combination of BRAF and MEK inhibitors, alternative BRAF and MEK inhibitors might be substituted. However, caution is warranted Figures 1–3Table 1.

**Table 1:**
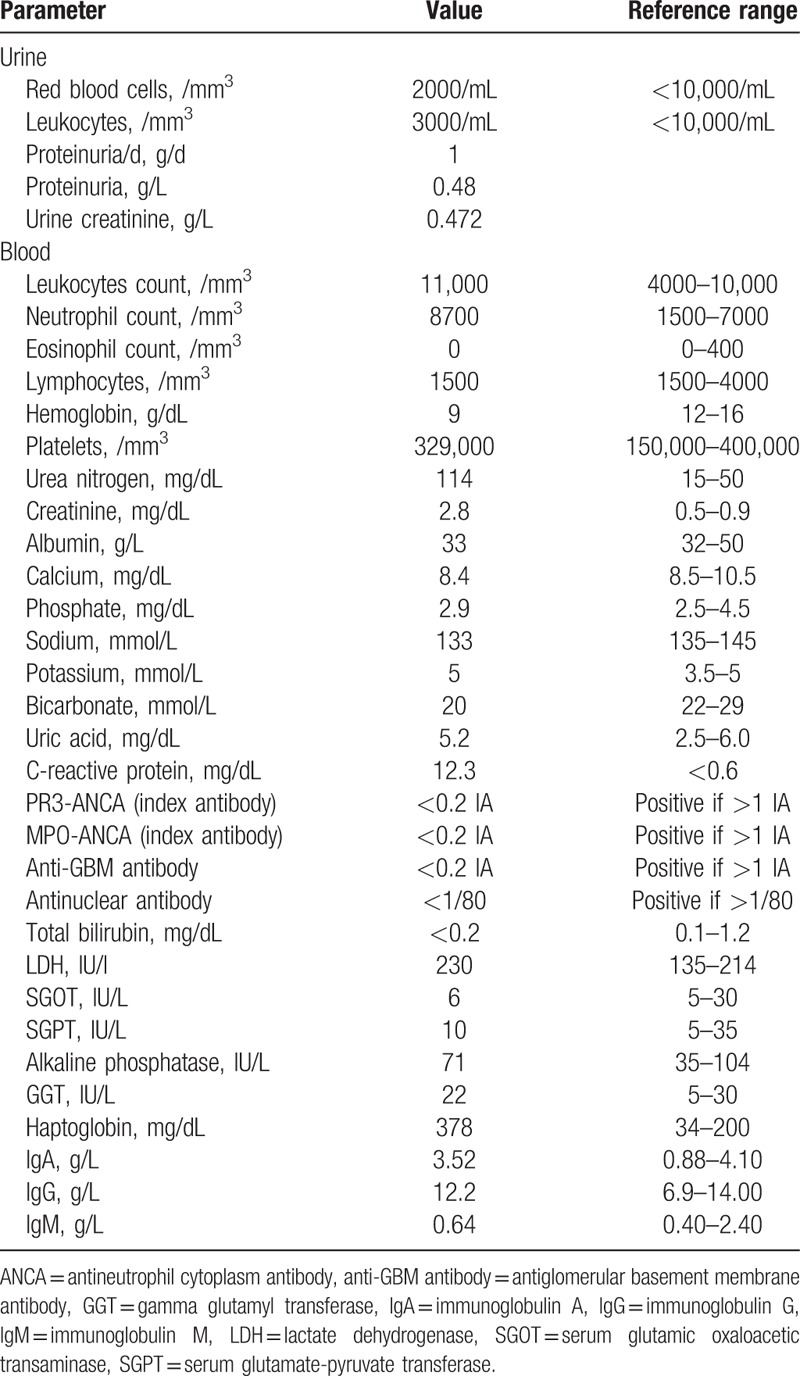
Laboratory results on admission.
